# Comparison of Two High-Dose Versus Two Standard-Dose Influenza Vaccines in Adult Allogeneic Hematopoietic Cell Transplant Recipients

**DOI:** 10.1093/cid/ciad458

**Published:** 2023-08-16

**Authors:** Lora D Thomas, Einas Batarseh, Lubna Hamdan, Zaid Haddadin, Daniel Dulek, Spyros Kalams, Laura S Stewart, Anna L Stahl, Herdi Rahman, Justin Z Amarin, Haya Hayek, Michael Ison, Edgar T Overton, Steven A Pergam, Andrew J Spieker, Natasha B Halasa, the Adult HCT Flu Study, B Savani, Kassim, M Logue, S Barto, R McHenry, S Tanna, L Bezler, P Al-Saden, M Marshall, D Salzman, A Greenstein, A Jackson, S Wright, M Flowers, M Loeffelholz, S Marquis, E Nguyen

**Affiliations:** Department of Medicine, Virginia Commonwealth University Medical Center, Richmond, Virginia, USA; Department of Pediatrics, Vanderbilt University Medical Center, Nashville, Tennessee, USA; Department of Pediatrics, Vanderbilt University Medical Center, Nashville, Tennessee, USA; Department of Pediatrics, Vanderbilt University Medical Center, Nashville, Tennessee, USA; Department of Pediatrics, Vanderbilt University Medical Center, Nashville, Tennessee, USA; Department of Pediatrics, Vanderbilt University Medical Center, Nashville, Tennessee, USA; 3Department of Medicine, Vanderbilt University Medical Center, Nashville, Tennessee, USA; Department of Pediatrics, Vanderbilt University Medical Center, Nashville, Tennessee, USA; Department of Pediatrics, Vanderbilt University Medical Center, Nashville, Tennessee, USA; Department of Pediatrics, Vanderbilt University Medical Center, Nashville, Tennessee, USA; Department of Pediatrics, Vanderbilt University Medical Center, Nashville, Tennessee, USA; Respiratory Disease Branch, Division of Microbiology and Infectious Diseases, National Institute of Allergy and Infectious Diseases, National Institutes of Health, Rockville, Maryland, USA; Department of Medicine, University Hospital, University of Alabama at Birmingham, Birmingham, Alabama, USA; Department of Medicine, Fred Hutchinson Cancer Center, Seattle, Washington, USA; Department of Biostatistics, Vanderbilt University Medical Center, Nashville, Tennessee, USA; Department of Pediatrics, Vanderbilt University Medical Center, Nashville, Tennessee, USA

**Keywords:** influenza vaccine, hematopoietic cell transplant

## Abstract

**Background:**

Adult hematopoietic cell transplant (HCT) recipients are at high risk for influenza-related morbidity and mortality and have suboptimal influenza vaccine immune responses compared to healthy adults, particularly within 2 years of transplant.

**Methods:**

This phase II, double-blind, multicenter randomized controlled trial compared 2 doses of high-dose trivalent (HD-TIV) to 2 doses of standard-dose quadrivalent (SD-QIV) influenza vaccine administered 1 month apart in adults 3–23 months post-allogeneic HCT. Hemagglutinin antibody inhibition (HAI) titers were measured at baseline, 4 weeks following each vaccine dose, and approximately 7 months post-second vaccination. Injection-site and systemic reactions were assessed for 7 days post-vaccination. The primary immunogenicity comparison was geometric mean HAI titer (GMT) at visit 3 (4 weeks after the second dose); we used linear mixed models to estimate adjusted GMT ratios (aGMRs) comparing HD-TIV/SD-QIV for each antigen.

**Results:**

We randomized 124 adults; 64 received SD-QIV and 60 received HD-TIV. Following the second vaccination, HD-TIV was associated with higher GMTs compared to SD-QIV for A/H3N2 (aGMR = 2.09; 95% confidence interval [CI]: [1.19, 3.68]) and B/Victoria (aGMR = 1.61; 95% CI: [1.00, 2.58]). The increase was not statistically significant for A/H1N1 (aGMR = 1.16; 95% CI: [0.67, 2.02]). There was a trend to more injection-site reactions for HD-TIV after the second vaccination compared to SD-QIV (50% vs 33%; adjusted odds ratio [aOR] = 4.53; 95% CI: [0.71, 28.9]), whereas systemic reactions were similar between groups with both injections.

**Conclusions:**

Adult allogeneic HCT recipients who received 2 doses of HD-TIV produced higher HAI antibody responses for A/H3N2 and B/Victoria compared with 2 doses of SD-QIV, with comparable injection-site or systemic reactions.

## Teaser

In this phase II, double-blind, multicenter randomized controlled trial of post-allogeneic hematopoietic cell transplant recipients, 2 doses of high-dose trivalent influenza vaccine had superior immunogenicity than 2 doses of standard-dose quadrivalent influenza vaccine.

## Graphical abstract

**Graphical abstract:**
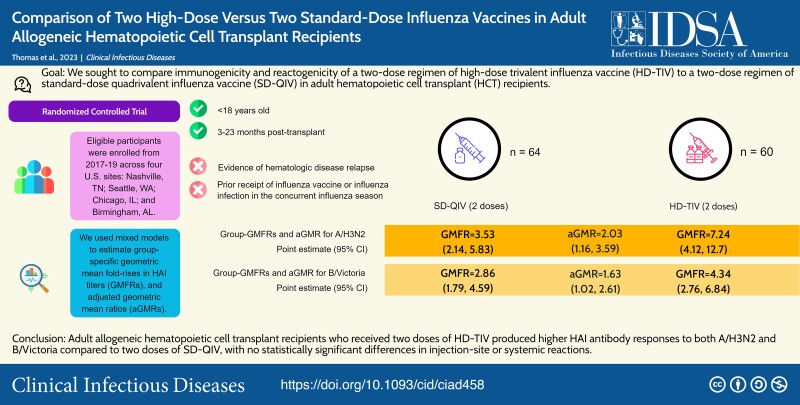
This graphical abstract is also available at Tidbit: https://tidbitapp.io/tidbits/comparison-of-two-high-dose-versus-two-standard-dose-influenza-vaccines-in-adult-allogeneic-hematopoietic-cell-transplant-recipients-17b01d1d-6b7c-486f-9645-5d38dbbfeabd

Hematopoietic cell transplant (HCT) recipients are at high risk for infection due to respiratory viruses, including influenza, particularly within the first 2 years post-HCT. Vaccination has been essential in the prevention of influenza-associated illness and reduction of influenza-related morbidity and mortality in adult HCT recipients. Prior studies of influenza vaccination in HCT recipients have noted poor immunogenicity compared to healthy controls, with seroconversion rates ranging from 13% to 59% after single-dose vaccination [1]. Despite their poor responses, the current guidelines recommend annual influenza vaccination after 3–6 months post-transplant [2, 3]. Multiple influenza vaccine studies in HCT recipients have noted improved immunogenicity for those who are later post-transplant; with less data about vaccine responses less than six months post-transplant [4–6]. Strategies to improve immunogenicity in HCT recipients are needed in order to establish optimal post-transplant vaccination regimens.

One alternative strategy is the administration of a high-dose inactivated influenza vaccine, which has been proven superior in an elderly population [7]. A single-center, phase I safety and immunogenicity study comparing one dose of high-dose trivalent influenza vaccine (HD-TIV) to standard-dose trivalent influenza vaccine (SD-TIV) in adult HCT recipients with a median of 7.9 months post-transplant reported higher geometric mean titers (GMT) for the A/H3N2 influenza strain compared SD-TIV, with no major safety concerns noted [8]. Another strategy is the administration of 2 standard doses of influenza vaccine in the same season, but prior studies of this strategy had small cohorts, with few participants in the early transplant period and did not compare 2 doses of HD to 2 doses of SD influenza vaccine [5, 9–12]. Therefore, we conducted a phase II, multicenter trial comparing 2 doses of HD-TIV to 2 doses of standard dose quadrivalent vaccine (SD-QIV) in adult HCT recipients.

## METHODS

### Trial Design and Participants

This was a prospective, multicenter, double-blinded, phase II, randomized controlled immunogenicity and safety trial comparing 2 doses of HD-TIV to 2 doses of SD-QIV in adult HCT recipients (ClinicalTrials.gov: NCT03179761). The trial was conducted during the 2017–18 and 2018–19 influenza seasons at 4 sites: Vanderbilt University Medical Center (Nashville, Tennessee, USA), which served as the leading site, Fred Hutchinson Cancer Center (Seattle, Washington, USA), Northwestern University (Chicago, Illinois, USA), and the University of Alabama at Birmingham (Birmingham, Alabama, USA).

Eligible participants were at least 18 years of age and 3–23 months post-allogeneic HCT. Participants with graft versus host disease (GVHD) were eligible if their disease and GVHD therapy were stable for at least 4 weeks prior to vaccination. Exclusion criteria included: hypersensitivity to influenza vaccination, eggs/egg protein, or latex; history of Guillain-Barre syndrome, current pregnancy, evidence of hematologic disease relapse, cirrhosis, human immunodeficiency virus infection; and prior receipt of influenza vaccine or documented influenza infection in the coinciding influenza season. Participants who had received a stem cell boost or delayed donor lymphocyte infusion within 90 days of enrollment or received immunoglobulin (Ig) therapy within 28 days of vaccination; and acute illness within 48 hours, receipt of any live vaccines within 4 weeks or any inactivated vaccines within 2 weeks prior to potential study vaccination were also excluded. Participants who required non-influenza vaccines while enrolled could receive these vaccines if administered at least 2 weeks prior to each study vaccine administered at visits 1 and 2.

Participants were randomized on a 1:1 basis to receive either 2 doses of the season-specific HD-TIV or SD-QIV, with a target interval of 28–42 days between vaccine doses (at the time of this study, the high-dose formulation of the quadrivalent vaccine was not available). Randomization, which occurred at visit 1 after eligibility criteria were met, was blocked and stratified by site and GVHD with systemic steroid use. Additional stratification was put in place for participants <12 months post-HCT by the following factors: alemtuzumab, anti-thymocyte globulin, cord blood transplant, haploidentical transplant, or post-transplant cyclophosphamide.

The study protocol was reviewed and approved by the Vanderbilt University Institutional Review Board (IRB), which served as the single IRB for all study sites. All participants provided written informed consent prior to conducting any study procedures. Study data were collected and managed using a REDCap database hosted at Vanderbilt.

### Vaccine

Vaccines were provided by Sanofi (Swiftwater, Pennsylvania, USA) and investigational pharmacies at each site dispensed study vaccines per randomization code. SD-QIV contained 15 µg of hemagglutinin from each strain (A/H1N1, A/H3N2, B/Victoria, B/Yamagata). HD-TIV contained 60 µg of hemagglutinin from each strain except for B/Yamagata (Supplementary Table 1).

### Study Procedures

Vaccines were administered as 0.5 mL intramuscular deltoid injections given at a target interval of 28–42 days apart (visits 1 and 2). Per protocol, complete blood count, CD4^+^/CD8^+^/CD19^+^ cells, total IgM and IgG concentrations, and blood for serological and cellular assays were scheduled for collection prior to administration of each vaccine dose, as well as 28–42 days (visit 3) following the second vaccine dose and 124–236 days (visit 4) following visit 3. Nasal swabs were obtained at each study visit.

### Safety Evaluations

Participants recorded injection-site and systemic reactions using a memory aid for 7 days after each vaccine. Reactions were graded according to a mild/moderate/severe toxicity scale (Supplementary Tables 5 and 6) and entered into REDCap. Grade 3 or higher unsolicited adverse events and severe adverse events (SAE) were also collected through seven days after each vaccination.

### Immunogenicity Assays

Serum samples were frozen at each site, shipped to Vanderbilt, and then bulk-shipped to Sanofi Global Clinical Immunology for blinded hemagglutination inhibition (HAI) testing for each vaccine-specific antigen [13]. When blood volume was insufficient, HAI testing of influenza A antigens was prioritized.

### Influenza Surveillance

Active influenza surveillance occurred during each site's local influenza season, defined as when ≥10% clinical or research laboratory samples tested positive for influenza for 2 consecutive weeks by either molecular or rapid testing [8, 14, 15]. During this period, weekly communication occurred, and a nasal swab was collected when a participant had influenza-like illness (ie, presence of fever and/or 2 of any of the following symptoms: respiratory symptoms [rhinorrhea, sinus congestion, post-nasal drip, shortness of breath, cough, wheezing, sputum production, sore throat, sneezing, watery eyes, ear pain, and hoarseness] or systemic symptoms [myalgias and headache]). Nasal specimens were shipped to Vanderbilt University Medical Center and tested using Luminex NxTAG RPP® plus influenza B lineage typing by singleplex polymerase chain reaction [16, 17].

### Statistical Analysis

Information regarding power calculations is available (Supplementary Table 2). Baseline descriptive statistics were reported as median (interquartile range [IQR]) for continuous variables and absolute and relative frequencies for categorical variables. All descriptive analyses were based on participants receiving at least 1 vaccine dose.

HAI titers to each antigen were summarized within each vaccine group at each visit as GMT, proportion with a titer ≥1:40 (a proxy for seroprotection), geometric mean fold-rise from baseline (GMFR: eg, HD-TIV visit 2 or 3/HD-TIV visit 1), and proportion with a ≥4-fold-rise from baseline (a proxy for seroconversion). The primary immunogenicity endpoints were the adjusted geometric mean ratios (aGMR) comparing the GMT between HD-TIV and SD-QIV following the second vaccine dose (visit 3). Superiority was considered to be achieved based on lower aGMR 95% confidence interval (CI) endpoints exceeding 1.0. No multiplicity adjustments were planned as the primary endpoints were pre-specified. Furthermore, B/Yamagata was analyzed as a control because this strain was included in SD-QIV but not in HD-TIV. The aGMR (HD-TIV/SD-QIV) was estimated using linear mixed models with log-transformed HAI titer, adjusting for age, log-transformed baseline titer, continuous time post-HCT, CD4^+^ count, CD19^+^ count, absolute lymphocyte count (ALC), and GVHD; and with participant- and site-specific random effects. We sought to identify predictors of visit 3 titers (28–42 days following the second vaccine dose) using a model analogous to the mixed model described above.

In all model-based analyses, missing data were addressed using multiple imputation by chained equations (M = 300 iterations). A total of 6 participants died during the post-vaccine follow-up period; their observations were included in analyses for as long as they were alive, though missing values for variables due to death were not imputed.

The primary safety endpoint (reactogenicity) was summarized as frequency of injection-site reactions (swelling, erythema, tenderness, and pain) and systemic reactions (fever [defined as ≥38.0°C], decreased activity, myalgia, nausea, headache, fatigue, and vomiting) within the 7-day periods following each vaccine dose. We analyzed reactogenicity outcomes using generalized linear mixed models (with a logistic link function, including subject- and site-specific random intercepts) to compare odds of adverse injection-site or systemic reactions separately following each dose.

## RESULTS

### Study Participants

**Figure 1.**
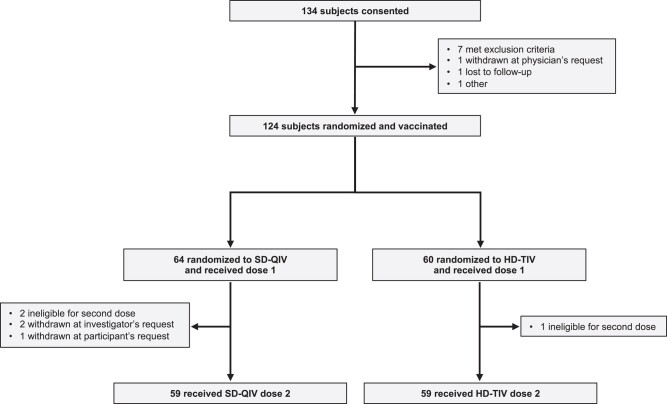
Enrollment, randomization, and vaccine status. A total of 134 participants were consented, among whom 124 were subsequently randomized and vaccinated. Among the 64 participants randomized to receive SD-QIV, 59 (92%) received both doses; among the 60 participants randomized to receive HD-TIV, 59 (98%) received both doses. Abbreviations: HD-TIV, high-dose trivalent; SD-QIV, standard-dose quadrivalent.

A total of 134 participants were enrolled; 124 were randomized, received at least 1 vaccine dose, and were considered evaluable for analysis (n = 64 received SD-QIV and n = 60 received HD-TIV, Figure 1). The median age was 57.8 years (IQR: [42.4, 64.1]), 48 (38.7%) were female, 111 (89.5%) were White, and 8 were (6.5%) Black; 3/123 (2.4%) were Hispanic/Latino. The median time post-HCT was 5.6 months (IQR: [3.7, 8.6]), 120 (96.8%) of HCTs were due to malignancy, and 57% received the first vaccine dose within 6 months of transplant. The overall cohort and each vaccination group had comparable demographic, clinical, and transplant characteristics (Table 1).

**Table 1.** Cohort Demographics and Clinical Characteristics, Further Stratified by Treatment Arm

	All (N = 124)	Control (SD-QIV) (N = 64)	Experimental (HD-TIV) (N = 60)
*Demographics*			
Age at enrollment, y			
Mean (SD)	52.7 (15.3)	56.8 (14.1)	48.4 (15.3)
Median (IQR)	57.8 (42.4, 64.1)	60.0 (51.4, 66.9)	52.7 (34.4, 61.6)
Minimum, maximum	18.5, 72.8	19.4, 72.8	18.5, 72.1
Gender, no. (%)			
Male	76 (61.3)	42 (65.6)	34 (56.7)
Female	48 (38.7)	22 (34.4)	26 (43.3)
Race, no. (%)			
White	111 (89.5)	60 (93.8)	51 (85.0)
Black/African American	8 (6.5)	3 (4.7)	5 (8.3)
Asian	1 (0.8)	0 (0)	1 (1.7)
Native Hawaiian	1 (0.8)	0 (0)	1 (1.7)
Other/unknown	3 (2.4)	1 (1.6)	2 (3.3)
Ethnicity, no. (%)			
Non-Hispanic	120/123 (97.6)	62/63 (98.4)	58 (96.7)
Hispanic	3/123 (2.4)	1/63 (1.6)	2 (3.3)
*Transplant characteristics, no. (%)*			
Reason for transplant			
Malignant	120 (96.8)	63 (98.4)	57 (95.0)
AML/ANLL	60/120 (50.0)	31/63 (49.2)	29/57 (50.9)
ALL	17/120 (14.2)	7/63 (11.1)	10/57 (17.5)
CML	6/120 (5.0)	4/63 (6.4)	2/57 (3.5)
MDS/MPN	20/120 (16.7)	13/63 (20.6)	7/57 (12.3)
Other	17/120 (14.2)	8/63 (12.7)	9/63 (14.3)
Non-malignant	4 (3.2)	1 (1.6)	3 (5.0)
Severe aplastic anemia	3/4 (75.0)	1/1 (100)	2/3 (66.7)
Other	1/4 (25.0)	0 (0)	1/3 (33.3)
Time from transplant to enrollment, mo			
Median (IQR)	5.6 (3.7, 8.6)	6.0 (3.6, 7.9)	5.2 (4.0, 9.5)
≥3 to <6 mo	71 (57.3)	33 (51.6)	38 (63.3)
≥6 to <12 mo	31 (25.0)	20 (31.3)	11 (18.3)
≥12 to <36 mo	22 (17.7)	11 (17.2)	11 (18.3)
Donor type			
Unrelated	71 (57.3)	35 (54.7)	36 (60.0)
Related	53 (42.7)	29 (45.3)	24 (40.0)
Stem cell source			
Bone marrow	19 (15.3)	8 (12.5)	11 (18.3)
Peripheral blood	98 (79)	54 (84.4)	44 (73.3)
Umbilical cord blood	7 (5.7)	2 (3.1)	5 (8.3)
Condition preparation regimen			
Myeloablative	59 (48.4)	30 (48.4)	29 (48.3)
Reduced-intensity or non-myeloablative	60 (49.2)	30 (48.4)	30 (50)
Total body irradiation	45 (39.1)	21 (36.2)	24 (42.1)
T-cell depletion	17 (14.4)	9 (14.8)	8 (14.0)
GVHD status at vaccine 1			
Acute	7 (5.7)	6 (9.4)	1 (1.7)
Chronic	28 (22.6)	16 (25.0)	12 (20.0)
Rituximab post-transplant	17 (13.7)	5 (7.85)	12 (20.0)
Recipient CMV status, negative	47 (37.9)	23 (35.9)	24 (40.0)
*Baseline lab values at visit 1 – median (IQR)*			
WBC (10^3^/μL)	5.1 (3.9, 6.4)	5.3 (4.3, 7)	5.1 (3.8, 6.2)
ANC (10^3^/μL)	3.3 (2.4, 4.3)	3.4 (2.6, 4.5)	3.1 (2.3, 4.2)
ALC (10^3^/μL)	1.0 (0.6, 1.5)	1.0 (0.5, 1.6)	0.9 (0.7, 1.3)
CD4^+a^ count	234 (137, 351)	232 (135, 335)	237 (152, 353)
CD8^+a^ count	306 (129, 601)	320 (145, 794)	263 (100, 469)
CD19^+b^ count	82 (17, 206)	87 (18, 220)	80 (17, 197)
Hemoglobin (g/dL)	12 (10.2, 13.2)	12 (9.9, 13.4)	12.1 (11, 13.2)
Platelets (10^3^/μL)	153 (111, 195)	150 (102, 194)	154 (120, 200)
Quantitative IgG^c^ (mg/dL)	633 (456, 887)	620 (409, 872)	643 (463, 903)
Quantitative IgM^c^ (mg/dL)	57 (29, 88)	55 (29, 86)	58 (30, 90)

Abbreviations: ALC, absolute leukocyte count; ALL, acute lymphoblastic leukemia; AML, acute myelogenous leukemia; ANC, absolute neutrophil count; ANLL, acute non-lymphocytic leukemia; CML, chronic myelogenous leukemia; GMFR, geometric mean fold-rise; HD-TIV, high-dose trivalent; IgG, immunoglobulin G; IgM, immunoglobulin M; IQR, interquartile range; MDS, myelodysplastic syndromes; MPN, myeloproliferative neoplasms; N, mumber of participants enrolled who received at least 1 vaccination; SD, standard deviation; SD-QIV, standard-dose quadrivalent; WBC, white blood count.

^a^CD4^+^ and CD8^+^ results missing for 3 SD-QIV subjects and 1 HD-TIV subject.

^b^CD19^+^ results missing for 3 SD-QIV subjects and 2 HD-TIV subjects.

^c^Quantitative IgG and IgM results missing for 1 SD-QIV subject and 1 HD-TIV subject.

### Immunogenicity Outcomes

**Figure 2.**
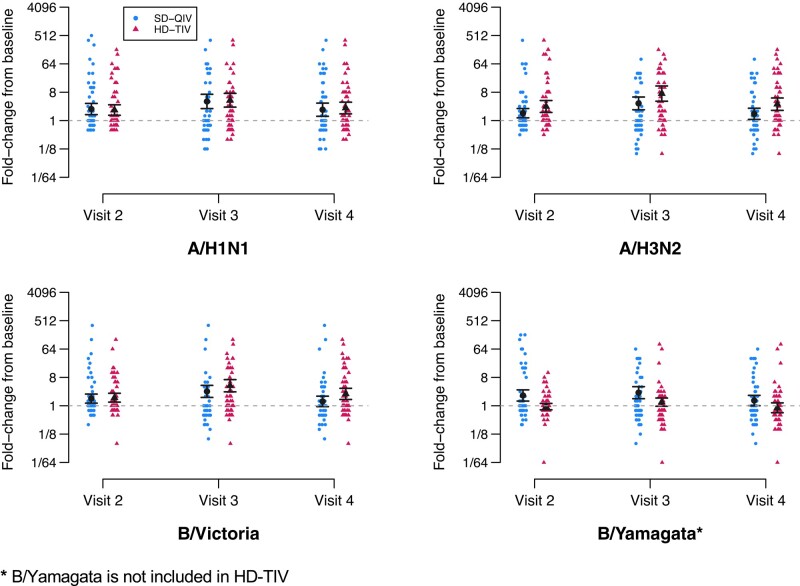
Fold-rises by vaccine group and dose. Depiction of titer fold-rises from baseline (visit 1, prior to the first dose), shown by randomization group (SD-QIV and HD-TIV) for each antigen and each follow-up visit. The estimated GMFR and 95% confidence intervals are depicted in black. Visit 2 titers are measured at a target window of 28–42 d following the first dose (prior to the second dose), visit 3 titers are measured at a target window of 28–42 d following the second dose, and visit 4 titers are measured at a target window of 124–236 d following the visit 3. Furthermore, B/Yamagata was not included in HD-TIV. Abbreviations: GMFR, geometric mean fold-rise; HD-TIV, high-dose trivalent; SD-QIV, standard-dose quadrivalent.

Significant increases in HAI antibody titers from baseline (ie, GMFRs, defined as follow-up/baseline for each group at each time) were noted at all time points for each vaccine group except for B/Yamagata strains in HD-TIV recipients (Figure 2, Table 2, Supplementary Table 3). Estimates and 95% CIs for GMFRs and aGMRs (HD-TIV/SD-QIV at each follow-up time) are provided in Table 2 for each vaccine group at each follow-up. Following the second dose (primary immunogenicity outcome), HD-TIV was associated with higher GMTs as compared to SD-QIV (aGMR HD-TIV/SD-QIV) for A/H3N2 (aGMR = 2.09; 95% CI: [1.19, 3.68]) and B/Victoria (aGMR = 1.61; 95% CI: [1.00, 2.58]). For B/Yamagata (analyzed as a control since this strain was not included in HD-TIV), the GMT was higher for SD-QIV (aGMR = 0.51 (95% CI: [0.33, 0.80]).

**Table 2.** Point Estimates and 95% CIs for Group-Specific Geometric Mean Fold-Rrises (GMFRs) and Adjusted Geometric Mean Ratios (aGMRs, Comparing High Dose [HD-TIV] to Standard Dose [SD-QIV]), Shown for Each Antigen at Each Follow-up Visit

	GMFR (95% CI)		aGMR (95% CI)
	SD-QIV (n = 64)	HD-TIV (n = 60)	(HD-TIV/SD-QIV)
A/H1N1			
Visit 2	**2.33 [1.54, 3.53]**	**2.17 [1.48, 3.18]**	1.06 [0.62, 1.82]
Visit 3	**4.08 [2.33, 7.14]**	**4.45 [2.67, 7.39]**	1.14 [0.65, 1.98]
Visit 4	**2.22 [1.28, 3.86]**	**2.51 [1.59, 3.97]**	1.07 [0.60, 1.89]
A/H3N2			
Visit 2	**1.72 [1.22, 2.43]**	**2.81 [1.82, 4.35]**	1.55 [0.90, 2.69]
Visit 3	**3.53 [2.14, 5.83]**	**7.24 [4.12, 12.7]**	**2.03 [1.16, 3.59]**
Visit 4	**1.64 [1.03, 2.62]**	**3.33 [2.05, 5.40]**	**1.87 [1.05, 3.34]**
B/Victoria			
Visit 2	**1.69 [1.21, 2.36]**	**1.80 [1.30, 2.50]**	1.10 [0.70, 1.75]
Visit 3	**2.86 [1.79, 4.59]**	**4.34 [2.76, 6.84]**	**1.63 [1.02, 2.61]**
Visit 4	1.37 [0.89, 2.13]	**2.39 [1.54, 3.69]**	**1.63 [1.00, 2.65]**
B/Yamagata ^a^			
Visit 2	**2.12 [1.40, 3.22]**	0.94 [0.74, 1.19]	**0.45 [0.30, 0.70]**
Visit 3	**2.61 [1.63, 4.18]**	1.30 [0.96, 1.76]	**0.52 [0.34, 0.81]**
Visit 4	1.47 [0.95, 2.26]	0.86 [0.59, 1.26]	**0.53 [0.33, 0.83]**

Visit 2 titers are measured at a target window of 28–42 days following the first dose (prior to the second dose), visit 3 titers are measured at a target window of 28–42 days following the second dose, and visit 4 titers are measured at a target window of 138–222 days following visit 3. Bolding indicates statistical significance at the 0.05 level (two-sided).

Abbreviations: CI, confidence interval; HD-TIV, high-dose trivalent; SD-QIV, standard-dose quadrivalent.

^a^B/Yamagata is not included in HD-TIV.

### Predictors of Post-dose 2 Antibody Titers

Covariate-specific aGMRs for predictors of HAI titers to A/H1N1, A/H3N2, and B/Victoria following the second dose are presented in Table 3. Baseline HAI titers were predictive of post-dose 2 titers for all 3 antigens. Additionally, the receipt of HD-TIV, longer time post-HCT, higher CD4^+^ and CD19^+^, and lower ALC cell counts at the time of enrollment were significantly associated with higher post-dose 2 titers for at least 1 antigen.

**Table 3.** Point Estimates and 95% CIs for aGMRs Associated With Each Model Covariate for Visit 3 (Post-Dose 2) HAI Titers to Influenza Antigens

	A/H1N1		A/H3N2		B/Victoria	
	aGMR	95% CI	aGMR	95% CI	aGMR	95% CI
HD-TIV	1.24	[0.67, 2.36]	**2.25**	**[1.20, 4.22]**	**1**.**60**	**[0.96, 2.67]**
log_2_-baseline titer	**1**.**23**	**[1.06, 1.43]**	**1**.**33**	**[1.16, 1.52]**	**1**.**25**	**[1.10, 1.41]**
Age (y)	1.00	[0.98, 1.02]	1.00	[0.98, 1.02]	0.99	[0.98, 1.01]
Time post-HCT (mo)	1.06	[0.99, 1.14]	**1**.**06**	**[1.00, 1.14]**	1.00	[0.95, 1.06]
CD4^+^ count	**1**.**21**	**[1.00, 1.47]**	1.14	[0.94, 1.40]	**1**.**18**	**[1.01, 1.39]**
CD19^+^ count	1.12	[0.96, 1.29]	**1**.**24**	**[1.07, 1.44]**	**1**.**25**	**[1.11, 1.40]**
ALC (100/μL)	0.96	[0.91, 1.02]	0.94	[0.89, 1.00]	**0**.**94**	**[0.90, 0.99]**
GVHD history	1.28	[0.64, 2.54]	0.83	[0.41, 1.71]	0.95	[0.53, 1.67]

Bolding indicates statistical significance at the 0.05 level (two-sided).

Abbreviations: AGMR, adjusted geometric mean ratio; ALC, absolute leukocyte count; CI, confidence interval; GVHD, graft versus host disease; HD-TIV, high-dose trivalent; SD-QIV, standard-dose quadrivalent.

### Durability of Vaccine Immunogenicity

At visit 4 (approximately six months after the visit 3), titers to all antigens included in HD-TIV (ie, A/H1N1, A/H3N2, and B/Victoria), were significantly higher as compared to baseline titers (Supplementary Table 3). On the other hand, recipients of SD-QIV had significantly higher titers from baseline for influenza A antigens only. For both vaccine groups, the estimated visit 4 GMFRs approximately resemble the estimated GMFRs associated with a single dose (ie, at visit 2). The geometric mean titer to A/H3N2 was significantly higher for HD-TIV as compared to SD-QIV at visit 4 (aGMR = 1.87; 95% CI: [1.05, 3.34]) and for B/Victoria (aGMR = 1.63; 95% CI: [1.00, 2.65]).

### Reactogenicity and Safety

**Figure 3.**
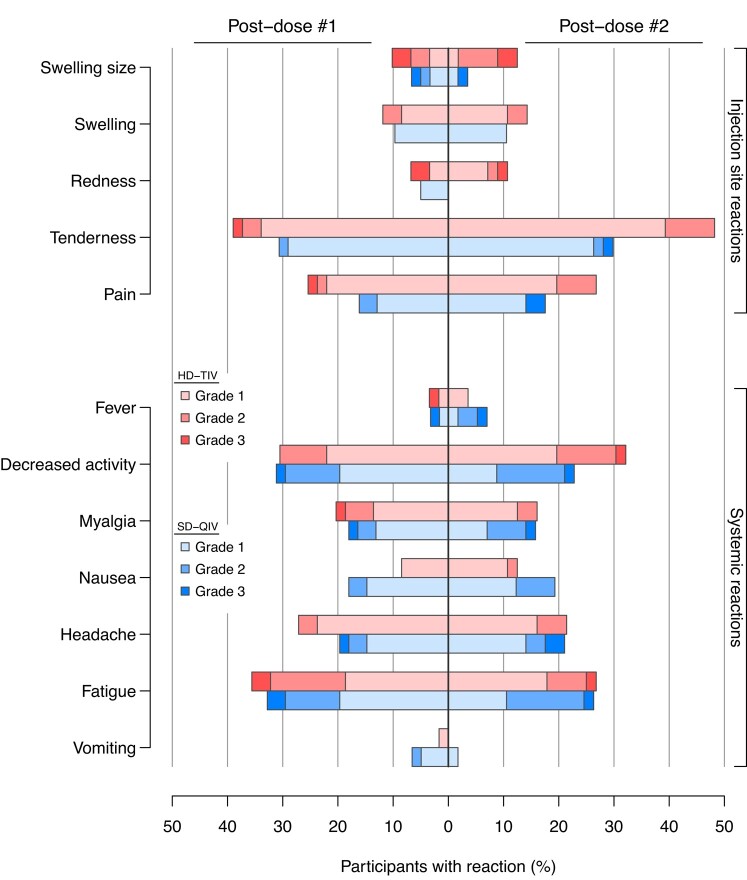
Injection-site and systemic reaction frequencies. Displayed are the relative frequencies of each injection site and systemic reaction type for each vaccine group (SD-QIV vs HD-TIV) following each dose. Reactions were further graded according to a mild/moderate/severe toxicity scale (grades 1 through 3, respectively), which are additionally marked by shading. Abbreviations: HD-TIV, high-dose trivalent; SD-QIV, standard-dose quadrivalent.

The most reported injection-site reactions after each vaccine dose for both groups were pain and tenderness (Figure 3, Supplementary Figure 1). The frequency of any injection-site reaction was higher for the HD-TIV group (49%) as compared to the SD-QIV (37%) following the first dose (adjusted odds ratio [aOR] = 3.44; 95% CI: [0.57, 20.7]), but not statistically significant. Similarly, the frequency of any injection-site reaction was higher, but also not statistically significant, for the HD-TIV (50%) compared to SD-QIV (33%) following the second dose (aOR = 4.53; 95% CI: [0.71, 28.9]). The frequency of any grade 3 (severe) injection-site reactions was 11% for HD-TIV and 7.0% for SD-QIV.

The most reported systemic reaction was fatigue for both groups (Figure 3, Supplementary Figure 2). No significant differences in systemic reactions were noted between groups following the first dose (45% for SD-QIV and 53% for HD-TIV; aOR = 1.34; 95% CI: [0.47, 3.81]) or the second dose (47% for SD-QIV and 46% for HD-TIV; aOR = 0.76; 95% CI: [0.26, 2.21]). The frequency of any grade 3 (severe) systemic reactions was similar between groups (7.1% for HD-TIV and 8.8% for SD-QIV).

### Laboratory Confirmed Influenza Cases

We identified a total of 7 individuals (5.6%) with laboratory-confirmed influenza infections; 5 cases in the HD-TIV group and 2 in the SD-QIV group (Supplementary Table 4). Two of the 5 cases in the HD-TIV group were due to B/Yamagata, which was not included in the HD-TIV, and the remaining 3 cases were A/H3N2. In the SD-QIV group, both cases were due to A/H3N2. No individuals diagnosed with influenza required hospitalization.

## DISCUSSION

This multicenter, double-blinded, phase II, randomized, controlled trial of 124 adult HCT recipients demonstrated that 2 doses of HD-TIV given at least 4 weeks apart was more immunogenic for influenza A/H3N2 and B/Victoria compared to 2 doses of SD-QIV, with higher GMTs 1 month after the second dose. Furthermore, the GMTs for A/H3N2 and B/Victoria were higher 6 months after the second dose in the HD-TIV group compared to the SD-QIV, signifying that the relative benefit of HD-TIV to SD-QIV is durable throughout the length of an influenza season. In addition, the safety profiles were comparable for both systemic reactions or injection-site reactions between groups. Notably, most injection-site reactions resolved within 2 days of vaccination. The increased immunogenicity and similar safety profiles are important findings as adult HCT recipients are at considerable risk for severe influenza disease and influenza-related complications. Thus, determining the optimal influenza vaccine strategy is essential.

Our study provides further support that a high-dose influenza vaccine strategy provides better immunogenicity than standard dose influenza vaccine. Our prior phase I, single-center study of 44 adult HCT recipients (median time post-HCT: 7.9 months) reported that a single dose of HD-TIV produced a higher GMT (GMR = 6.9) and a higher percentage of individuals with protective titers to A/H3N2 (81% vs 36%) compared to a single dose of SD-TIV [8]. Additionally, these results are consistent with a prior phase II trial of 161 adult solid organ transplant recipients, in which HD-TIV was associated with higher GMTs as compared to SD-TIV for all 3 antigen strains [18]. These findings are further consistent with our pediatric HCT trial of 170 participants, in which we found that 2 doses of HD-TIV resulted in higher antibody responses to both influenza A antigens as compared to 2 doses of SD-QIV [19]. Collectively, these data suggest HD-IIV is a practical strategy to overcome suboptimal immune responses in these vulnerable populations.

Our study is unique in that it compared 2 doses of HD-TIV to SD-QIV in an adult HCT population and found that 2 doses of either was associated with higher GMTs after each dose compared to baseline. Furthermore, the HD-TIV group met each of the 3 criteria for the historical World Health Organization biological standards for influenza vaccines after 2 doses for all 3 antigens: (1) >40% achieving seroconversion (4-fold-rise), (2) a GMFR from baseline of >2.5, and (3) >70% achieving seroprotection (HAI titer ≥1:40). The SD-QIV group did not meet the criteria for seroconversion (Supplementary Table 3). In a prior phase III study in the elderly comparing a single dose of HD-TIV to a single dose of SD-TIV, a superiority GMR benchmark of 1.5 was needed for licensure [20]. This benchmark (ie, aGMR comparing HD-TIV to SD-QIV) was met for both A/H3N2 (aGMR: 2.03) and B/Victoria (aGMR: 1.63) after 2 doses in our HD-TIV group. The previous studies evaluating methods to improve vaccine immunogenicity in HCT recipients have primarily focused on 2 doses of standard influenza vaccine administered within the same influenza season [5, 10–12], In these studies, 2 doses of influenza vaccine had variable effects on the seroresponse rate in HCT recipients compared to a single dose. A study evaluating immunogenicity in HCT recipients who received 2 doses of the ASO3-adjuvanted influenza A/H1N1 vaccine showed that seroconversion rates improved from 54% after the first dose, to 84% after the second dose [21]. This study also noted that those individuals who were <12 months from transplant exhibited a serological response rate of 21%. However, ASO3-adjunvanted influenza vaccines are not available universally; therefore, administration of 2 HD-IIV-dose strategy could be implemented readily.

Our study is also distinct from prior influenza vaccine studies of immunocompromised hosts by the fact we followed our cohort at least 6 months following their second dose to assess durability of immunogenicity. This study demonstrated that HD-TIV HAI titers were higher compared to baseline for at least 6 months following the completion of a 2-dose regimen for all 3 antigens. This study also demonstrated that the relative benefit of HD-TIV compared to SD-QIV was also sustained long-term for 2 out of 3 antigens (in particular, A/H3N2 and B/Victoria). These findings are particularly compelling because over half of the participants were vaccinated between 3 and 6 months post-transplant. This provides further evidence favoring a 2-dose regimen of HD influenza vaccine in this high-risk population, including in the early transplant period.

This study also demonstrated that adult HCT recipients tolerated HD-TIV and grade 3 reactions were infrequent. These findings are similar to what has been observed in our previous phase I studies comparing 1 dose HD-TIV to 1 dose SD-TIV in immunocompromised populations [8, 14]. Collectively, the prior phase I and phase II trials in both pediatric and adult immunocompromised hosts provide sufficient evidence that HD influenza vaccines are safe in these high-risk populations [18, 22–24].

This study is subject to limitations. We did not include a non-immunocompromised adult control group. Importantly, the HD-TIV product used in this trial did not include B/Yamagata; however, HD-QIV is now licensed. The study was conducted over 2 years and the specific antigen strains for A/H3N2 and B/Victoria were different between the 2 seasons. Even though active influenza surveillance was conducted, this trial was not powered to determine the efficacy of HD-TIV compared to SD-QIV in preventing influenza infection in this population, but the cases of influenza due to vaccine strains were similar between both groups.

## CONCLUSION

This study found that a 2-dose regimen of HD-TIV was associated with greater immunogenicity as compared to SD-QIV in adult HCT recipients and higher titers in the HD-TIV group were maintained over the entire influenza season. Furthermore, both vaccine regimens were well tolerated. Data from this study provide evidence to support implementation of a 2-dose regimen of HD inactivated influenza vaccine in this high-risk population.

## Supplementary Data

Supplementary materials are available at *Clinical Infectious Diseases* online. Consisting of data provided by the authors to benefit the reader, the posted materials are not copyedited and are the sole responsibility of the authors, so questions or comments should be addressed to the corresponding author.

## Supplementary Material

ciad458_Supplementary_Data

## Notes

***Author contributions.*** Designed research: N. B. H., L. D. T., L. S. S., M. I., S. A. P., E. T. O., A. J. S., S. K. Performed research, recruitment, acquisition: L. D. T., E. B., L. H., Z. H., D. D., S. K., L. S. S., A. L. S., M. I., E. T. O., S. A. P., N. B. H. Manuscript drafting: L. D. T., E. B., N. B. H., J. Z. A., H. H., A. J. S., A. L. S., H. R. Manuscript reviewing: M. I., E. T. O., S. A. P. Statistical analysis: A. J. S., A. L. S., H. R. Administrative: N. B. H., L. S. S., H. R., A. L. S.

***Adult HCT Flu Study*.** VUMC: B. Savani, Kassim, M. Logue, S. Barto, R. McHenry. NW: S. Tanna, L. Bezler, P. Al-Saden, M. Marshall. UAB: D. Salzman, A. Greenstein, A. Jackson, S. Wright. FHCC: M. Flowers, M. Loeffelholz, S. Marquis, E. Nguyen.

***Acknowledgments.*** VUMC: M. Dunn, B. Szeles, E. Yepsen, C. Guevara Pulido. NW: M. Angarone, K. Krueger. UAB: A. Peinhardt, M. Garner, B. Ratliff, K. Safavy, H. Dowling, C. Chapleau. The authors thank the patients, the HCT teams, and their data safety monitoring board.

***Financial support*.** S. A. P., S. K., and L. D. T. report that National Institute of Allergy and Infectious Diseases (NIAID)/National Institutes of Health (NIH) provided funding for this study (grant number 1U01AI132004). This work was also supported by Clinical and Translational Science Awards (award number UL1TR002243). L. S. S. reports grant support from NIH. N. B. H. reports grant support from NIH and healthcare-associated infections (HAI) testing and vaccine donation from Sanofi. A. J. S. reports support from NIH.
