# Supplementary material for: Comparison of Two High-Dose Versus Two Standard-Dose Influenza Vaccines in Adult Allogeneic Hematopoietic Cell Transplant Recipients
Source: Clin Infect Dis. 2023 Aug 16;77(12):1723–32. doi: 10.1093/cid/ciad458 (PMC10724468; doi:10.1093/cid/ciad458)

**SUPPLEMENTARY APPENDIX**

**Supplementary Methods:**

**Table S1.** Influenza vaccine strains by study season.

| **Season** | **A/H1N1** | **A/H3N2** | **B/Victoria** | **B/Yamagata*** |
| --- | --- | --- | --- | --- |
| **2017-2018** | A/Michigan/45/2015 | A/Hong Kong/4801/2014 | B/Brisbane/60/2008 | B/Phuket/3073/2013 |
| **2018-2019** | A/Michigan/45/2015 | A/Singapore/INFIMH-160019/2016 | B/Colorado/06/2017 | B/Phuket/3073/2013 |

- HD-TIV (Fluzone^®^ High Dose, Sanofi Pasteur) contained 60µg of H1N1, H3N2, and B/Victoria strains and SD-QIV (Fluzone^®^ Quadrivalent, Sanofi Pasteur) contained 15µg of all four strains.

| **Table S2. Sample Size and Power Calculations.** Our study’s power calculations were based on an analysis of a difference in proportions of participants in each vaccine group achieving a titer ≥1:40. We determined that between total of 80 to 110 evaluable participants (40 or 55 in each group) would be necessary to achieve 80% power and that 25-30% of the subjects in the SD group will achieve a protective titer compared to 50-60% of the HD-TIV group for at least one influenza antigen. | | | | | | |
| --- | --- | --- | --- | --- | --- | --- |
| Strain | Expected  Seroprotection HD-TIV | Expected  Seroprotection SD-QIV | N (HD) | N (SD) | Power | Total N with  20%  drop out |
| **Expected HD-TIV seroprotection is twice as of SD-QIV** | | | | | | |
| A | 40% | 20% | 80 | 80 | 80% | 200 |
| A | 50% | 25% | 55 | 55 | 80% | 138 |
| A | 60% | 30% | 40 | 40 | 80% | 100 |
| A | 70% | 35% | 28 | 28 | 80% | 70 |

**Table S3**.

Point estimates and 95% CIs for geometric mean HAI titer (GMT), titer ≥1:40, and proportion with ≥4-fold rise from baseline (not applicable for visit 1) for each vaccine regimen (SD-QIV and HD-TIV), stratified for each antigen. Also included is the ratio of the observed values (n) to the number of evaluable subjects (N) at each visit. Visit 1 titers measured at baseline (prior to first vaccine dose), visit 2 titers are measured at a target window of 28-42 days following the first dose (prior to the second dose), visit 3 titers are measured at a target window of 28-42 days following the second dose, and visit 4 titers are measured at a target window of 138-222 days following the second dose.

|  |  | A/H1N1 | | A/H3N2 | | B/Victoria | | B/Yamagata | |
| --- | --- | --- | --- | --- | --- | --- | --- | --- | --- |
| Visit |  | SD-QIV (N=64) | HD-TIV (N=60) | SD-QIV (N=64) | HD-TIV (N=60) | SD-QIV (N=64) | HD-TIV (N=60) | SD-QIV (N=64) | HD-TIV (N=60) |
| 1 | n/N (%) | 64/64 (100%) | 60/60 (100%) | 64/64 (100%) | 60/60 (100%) | 64/64 (100%) | 60/60 (100%) | 64/64 (100%) | 60/60 (100%) |
|  | GMT | 29 [20, 41] | 33 [23, 49] | 34 [23, 50] | 29 [19, 45] | 66 [48, 91] | 61 [42, 89] | 91 [66, 125] | 83 [57, 121] |
|  | % ≥1:40 | 42% [31%, 54%] | 40% [28%, 53%] | 48% [36%, 61%] | 42% [30%, 54%] | 69% [57%, 79%] | 68% [56%, 79%] | 77% [65%, 86%] | 77% [65%, 86%] |
| 2 | n/N (%) | 64/64 (100%) | 60/60 (100%) | 64/64 (100%) | 60/60 (100%) | 64/64 (100%) | 60/60 (100%) | 64/64 (100%) | 60/60 (100%) |
|  | GMT | 69 [45, 107] | 73 [47, 113] | 59 [38, 92] | 82 [50, 134] | 111 [79, 155] | 111 [72, 171] | 193 [130, 285] | 78 [53, 114] |
|  | % ≥1:40 | 59% [46%, 70%] | 62% [49%, 73%] | 60% [48%, 72%] | 65% [52%, 76%] | 79% [68%, 88%] | 73% [61%, 83%] | 87% [78%, 94%] | 72% [60%, 82%] |
|  | % ≥4-fold rise | 25% [16%, 37%] | 23% [14%, 35%] | 19% [11%, 30%] | 32% [21%, 44%] | 17% [9%, 28%] | 25% [15%, 37%] | 22% [13%, 33%] | 7% [2%, 15%] |
| 3 | n/N (%) | 60/64 (94%) | 59/60 (98%) | 60/64 (94%) | 59/60 (98%) | 60/64 (94%) | 59/60 (98%) | 60/64 (94%) | 59/60 (98%) |
|  | GMT | 119 [73, 194] | 148 [93, 237] | 106 [67, 168] | 210 [123, 356] | 171 [116, 253] | 265 [177, 397] | 218 [142, 334] | 108 [74, 157] |
|  | % ≥1:40 | 73% [61%, 84%] | 75% [63%, 84%] | 73% [61%, 84%] | 78% [66%, 87%] | 86% [75%, 93%] | 92% [83%, 97%] | 88% [77%, 94%] | 80% [68%, 89%] |
|  | % ≥4-fold rise | 39% [27%, 52%] | 41% [29%, 53%] | 38% [26%, 51%] | 49% [37%, 62%] | 29% [18%, 41%] | 44% [32%, 57%] | 27% [16%, 39%] | 10% [4%, 20%] |
| 4 | n/N (%) | 56/64 (88%) | 57/60 (95%) | 56/64 (88%) | 57/60 (95%) | 56/64 (88%) | 57/60 (95%) | 56/64 (88%) | 57/60 (95%) |
|  | GMT | 62 [39, 99] | 74 [47, 115] | 49 [30, 78] | 82 [51, 133] | 83 [56, 123] | 135 [90, 203] | 129 [89, 187] | 66 [46, 94] |
|  | % ≥1:40 | 65% [51%, 78%] | 66% [53%, 78%] | 49% [35%, 63%] | 66% [53%, 78%] | 73% [60%, 84%] | 83% [71%, 91%] | 84% [72%, 92%] | 64% [51%, 76%] |
|  | % ≥4-fold rise | 35% [22%, 49%] | 34% [22%, 47%] | 27% [16%, 40%] | 40% [27%, 53%] | 18% [9%, 31%] | 34% [22%, 47%] | 22% [12%, 35%] | 9% [3%, 19%] |

**Table S4**. Confirmed influenza positive cases following vaccination.

| **Year Group** | **Vaccine Dosage** |  | **Days since Dose 1** | **Days since Dose 2** |
| --- | --- | --- | --- | --- |
| 1 | HD-TIV | B/Yamagata^*^ | 131 | 96 |
| 1 | HD-TIV | B/Yamagata | 52 | 24 |
| 1 | SD-QIV | A/H3N2 | 52 | 11 |
| 2 | HD-TIV | A/H3N2 | 70 | 42 |
| 2 | HD-TIV | A/H3N2 | 14 | N/A |
| 2 | SD-QIV | A/H3N2 | 146 | 115 |

*****B/Yamagata is not included in HD-TIV.

Note “N/A” means swab was collected prior to the second dose of the study vaccine.

**Table S5.** Local solicited adverse events

| **Local Reaction** | **Mild (Grade 1)** | **Moderate (Grade 2)** | **Severe (Grade 3)** |
| --- | --- | --- | --- |
| Pain | Subject is aware of pain but it does not interfere with daily activity and no pain medication is taken | Subject is aware of pain; there is interference with daily activity or it requires therapeutic measures | Subject is aware of pain and it prevents daily activity or requires a healthcare visit |
| Tenderness | The area immediately surrounding the injection site hurts only when touched and it does not interfere with daily activity | The area immediately surrounding the injection site hurts only when touched and it interfere with daily activity | The area immediately surrounding the injection site hurts when touched and it prevents daily activity |
| Swelling/Induration | Does not interfere with daily activity | Interferes with daily activity | Prevents daily activity |
| Erythema/Redness | 0.5 to 2.5 cm | >2.5 cm to <5 cm | ≥5 cm |
| Induration/Swelling | 0.5 to 2.5 cm | >2.5 cm to <5 cm | ≥5 cm |

**Table S6.** Systemic solicited adverse events

| **Systemic (Subjective)** | **Mild (Grade 1)** | **Moderate (Grade 2)** | **Severe (Grade 3)** |
| --- | --- | --- | --- |
| Fatigue/Malaise | No interference with activity | Some interference with activity | Incapacitating, prevent s daily activity, may necessitate medical care or absenteeism |
| Headache | No interference with activity | Some interference with activity | Incapacitating, prevents daily activity, may necessitate medical care or absenteeism |
| Nausea | No interference with activity | Some interference with activity | Incapacitating, prevents daily activity, may necessitate medical care or absenteeism |
| Body ache/myalgia (not at injection site) | Less active than normal without interference with essential daily tasks (e.g. eating, sleeping) | Less active than normal with interference with essential daily tasks (e.g. eating, sleeping) | Incapacitating, prevents daily activity, may necessitate medical care or absenteeism |
| General activity level | Less active than normal without interference with essential daily tasks (e.g. eating, sleeping) | Less active than normal with interference with essential daily tasks (e.g. eating, sleeping) | Incapacitating, prevents daily activity, may necessitate medical care or absenteeism |
| Vomiting | 1-2 times a day | 3-4 times a day | >4 times a day |
| **Systemic (Quantitative)** | **Mild (Grade 1)** | **Moderate (Grade 2)** | **Severe (Grade 3)** |
| **Fever (°C) – ORAL** | ≥38.0 - <38.3  ≥100.4 < 101° F | ≥38.3 - <39  ≥ 101 - < 102° F | ≥39  ≥ 102° F |

**SUPPLEMENTARY FIGURE LEGENDS**

**Figure S1**. Injection site reaction frequencies by day post-vaccine dose. Displayed are the relative frequencies of any local reaction by day for each vaccine group (SD-QIV vs. HD-TIV) following each dose. Reactions were further graded according to a mild/moderate/severe toxicity scale (grades 1 through 3, respectively), which are additionally marked by shading.

**Figure S2**. Systemic reaction frequencies by day post-vaccine dose. Displayed are the relative frequencies of any severe reaction by day for each vaccine group (SD-QIV vs. HD-TIV) following each dose. Reactions were further graded according to a mild/moderate/severe toxicity scale (grades 1 through 3, respectively), which are additionally marked by shading.

**Figure S1**.


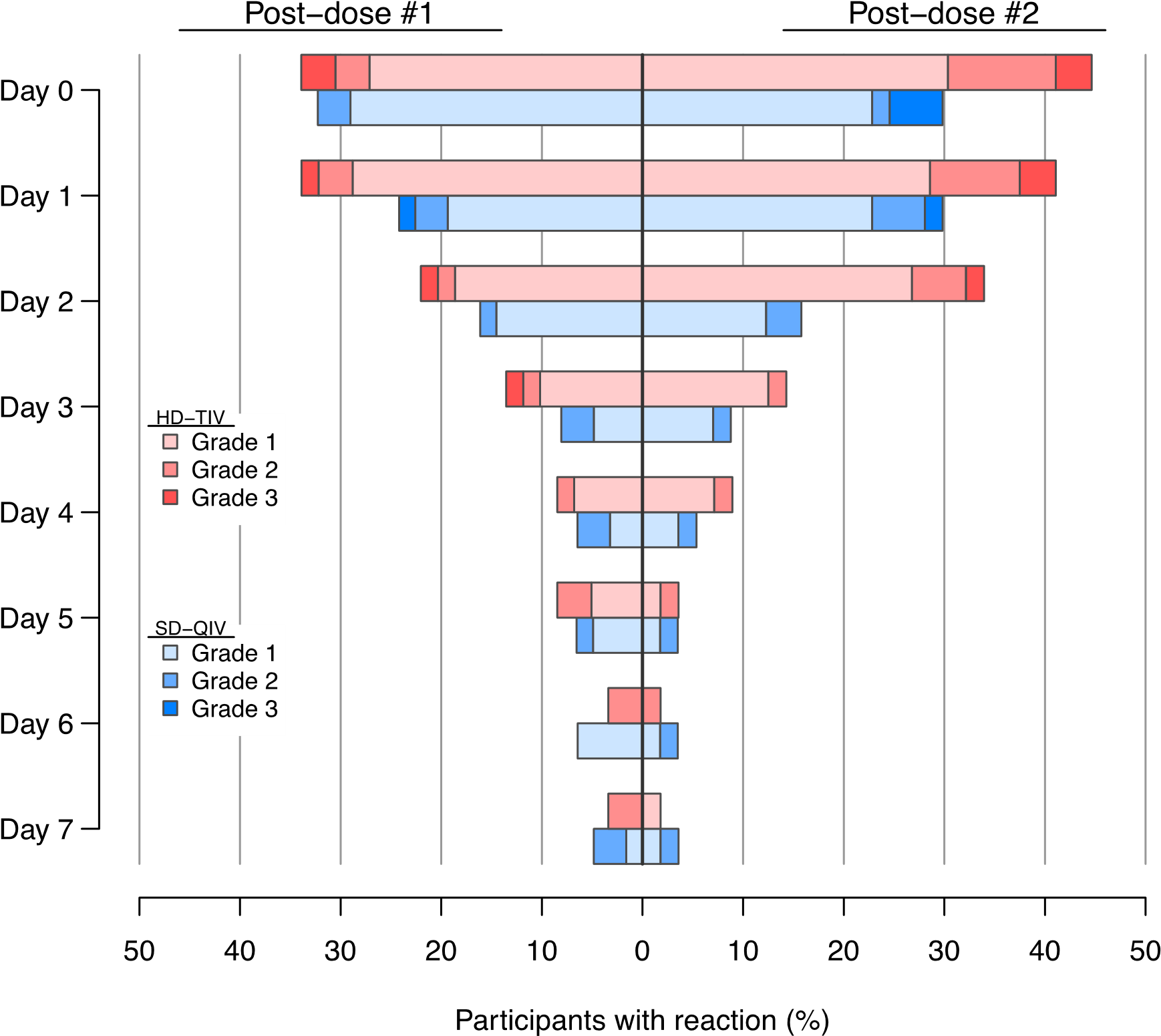


**Figure S2**.


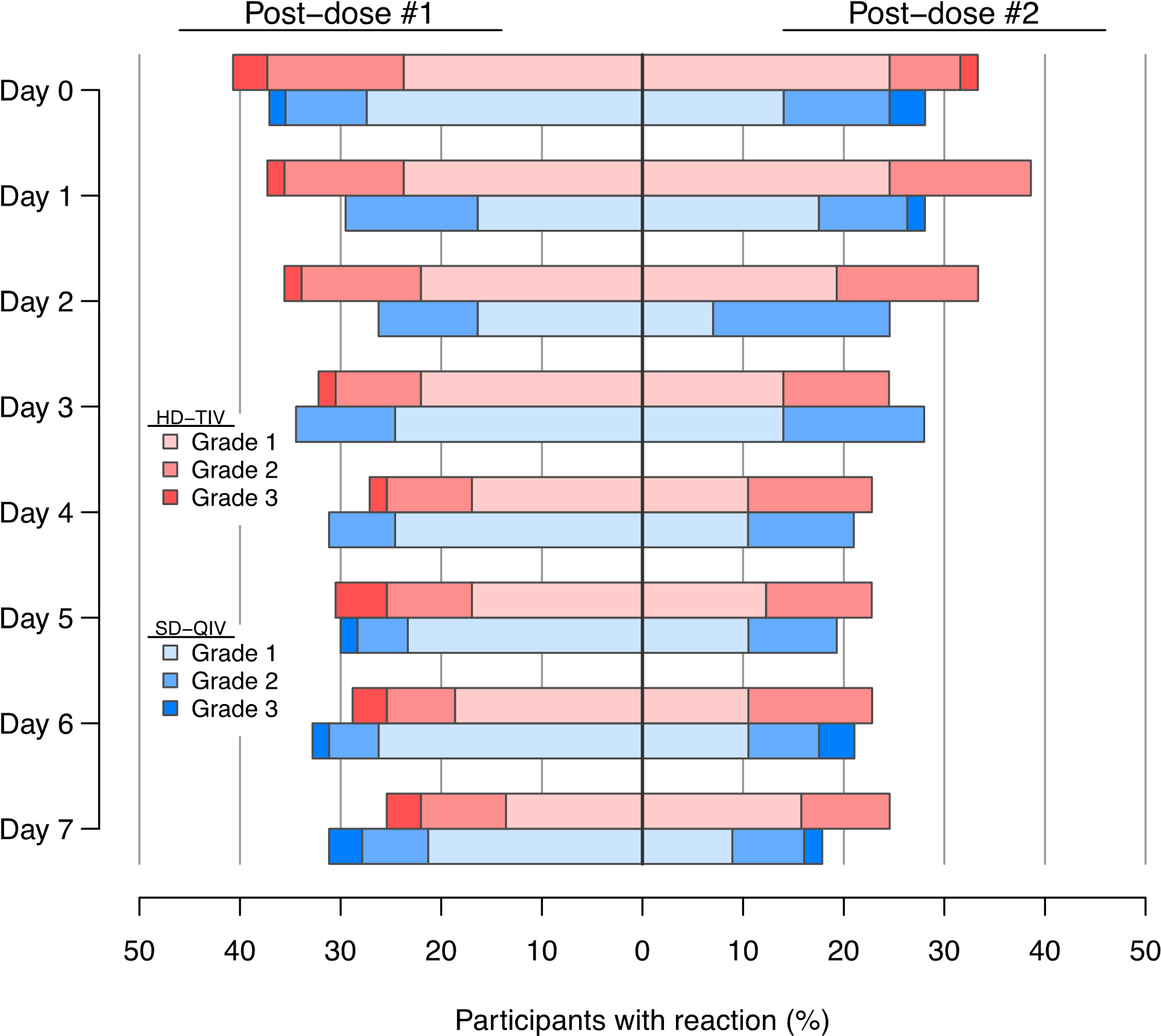

Supplement: ciad458_Supplementary_Data [file ciad458_supplementary_data.docx]
